# Hepatitis A, B and C viral co-infections among HIV-infected adults presenting for care and treatment at Muhimbili National Hospital in Dar es Salaam, Tanzania

**DOI:** 10.1186/1471-2458-8-416

**Published:** 2008-12-19

**Authors:** Tumaini J Nagu, Muhammad Bakari, Mecky Matee

**Affiliations:** 1 Department of Internal Medicine, School of Medicine, Muhimbili University of Health and Allied Sciences, P.O. Box 65001, Dar es Salaam, Tanzania; 2 Department of Microbiology and Immunology, School of Medicine, Muhimbili University of Health and Allied Sciences, P.O. Box 65347, Dar es Salaam, Tanzania

## Abstract

**Background:**

Tanzania is currently scaling-up access to anti-retro viral therapy (ART) to reach as many eligible persons as possible. Hepatitis viral co-infections are known to influence progression, management as well as outcome of HIV infection. However, information is scarce regarding the prevalence and predictors of viral hepatitis co-infection among HIV-infected individuals presenting at the HIV care and treatment clinics in the country.

**Methods:**

A cross-sectional study conducted between April and September 2006 enrolled 260 HIV-1 infected, HAART naïve patients aged ≥18 years presenting at the HIV care and treatment clinic (CTC) of the Muhimbili National Hospital (MNH). The evaluation included clinical assessment and determination of CD4+ T-lymphocyte count, serum transaminases and serology for Hepatitis A, B and C markers by ELISA.

**Results:**

The prevalence of anti HAV IgM, HBsAg, anti-HBc IgM and anti-HCV IgG antibodies were 3.1%, 17.3%, 2.3% and 18.1%, respectively. Dual co-infection with HBV and HCV occurred in 10 individuals (3.9%), while that of HAV and HBV was detected in two subjects (0.8%). None of the patients had all the three hepatitis viruses. Most patients (81.1%) with hepatitis co-infection neither had specific clinical features nor raised serum transaminases. History of blood transfusion and jaundice were independent predictors for HBsAg and anti-HBc IgM positivity, respectively.

**Conclusion:**

There is high prevalence of markers for hepatitis B and C infections among HIV infected patients seeking care and treatment at MNH. Clinical features and a raise in serum alanine aminotransferase were of limited predictive values for the viral co-infections. Efforts to scale up HAART should also address co-infections with Hepatitis B and C viruses.

## Background

The National HIV/AIDS Care and Treatment Plan (NCTP) for Tanzania Mainland launched in 2004 was charged with the responsibility of providing quality human immunodeficiency virus (HIV) care and treatment services to as many HIV-infected residents of the United Republic of Tanzania as possible. The operational plan was to provide antiretroviral treatment to as many 440,000 AIDS patients by the end of 2008; and to track the disease progression in some 1.2 million HIV-infected persons who were not clinically eligible for ART. The plan recommends that treatment be available through care and treatment clinics (CTC's) established over a five years time, at virtually all public and non-public hospitals down to the district level [1]. By December 2007, the numbers of health facilities providing HIV care and treatment was 210. These provided non-ART care to estimated 118,286 clients and ART to 69,250 eligible patients [2].

Despite these achievements, relatively little is known regarding hepatitis viral co-infections among HIV infected patients enrolling at the CTC's. To the best of our knowledge, this is the first study to determine the prevalence and predictors of viral hepatitis co-infection among adults infected with HIV attending a CTC in Tanzania. Thus, findings obtained will provide valuable information for stakeholders within and outside Tanzania involved in scaling up care and treatment services.

## Methods

### Study design and setting

This was a hospital based descriptive cross-sectional study conducted at the Muhimbili National Hospital's (MNH) HIV care and treatment centre (CTC) between April 2006 and September 2006. MNH is a public and tertiary hospital located in Dar es Salaam, the largest commercial city of Tanzania.

### Study population

Participants were the recently diagnosed HIV infected patients who were antiretroviral therapy (ART)-naïve, referred to the MNH-CTC from the various voluntary counselling and testing (VCT) centres in Dar es Salaam as well as the medical wards of MNH for initial work-up and possible initiation of treatment with antiretroviral therapy (ART).

### Interviews and clinical examination

Interviews were conducted using a structured standard questionnaire to obtain information regarding demographic characteristics, history of jaundice, and symptoms of flu-like illness such as fever, nausea and vomiting; duration of illness, past medical history of blood transfusion, traditional uvulectomy, scarification, use of parenteral illicit/recreational drugs, as well as sexual, family and social histories. Clinical examination, conducted according to standard clinical examination methods [3], and the subsequent staging of patients using the WHO HIV staging criteria [4], followed the interviews.

### Laboratory investigations

#### Specimens

Blood was collected aseptically into 10 ml vacutainer tubes (BD, NJ USA) for biochemical, CD4+ count and viral serology tests. Biochemical and CD4+ T lymphocyte assays were performed within three hours of collection, while serum for serological assays of hepatitis A, B and C markers was stored at -20°C until the time for assay.

#### CD4+ T-Lymphocytes enumeration

This was determined by flow cytometry using Becton Dickson Facs Calibur machine.

#### Alanine aminotransferase (ALAT) assay

The serum aminotransferase determined was alaninie aminotransferase (ALAT). The catalytic activity of ALAT (EC 2.6.1.2) was determined in serum using a COBAS MIRA chemistry analyzer (GMI, MI, USA) after it was calibrated.

#### Detection of anti HAV IgM, HBsAg, anti HbcAb IgM and Total anti HCV antibodies

The determination of anti hepatitis A virus antibody (anti HAV IgM), hepatitis B surface antigen (HBsAg), anti hepatitis B core IgM antibody (anti HbcAb-IgM), and anti hepatitis C virus IgG antibody (anti HCV) was done using the antibody capture ELISA (Adaltis – EIAgen kit). Samples that had reactivity to HCV were subjected to second ELISA (Abbott, USA), third-generation ELISA assay. Samples were considered to be positive for HCV if were reactive for both ELISAs.

#### Data handling and statistical analysis

Data was processed and analysed with SPSS version 12.0 [5]. The seroprevalence of HAV, HCV and HBV were expressed in percentages with their corresponding 95% CIs. Pearson's Chi-Square (χ^2^) was used to determine the association between the independent variables, however, where the numbers in a cell was less than 5, a Fisher's exact test was used instead. To approach normality, the baseline absolute CD4+ cell count data was log-transformed and Students' t-test was used to examine the association between the level of immunosuppression and presence of HAV, HBV and HCV infections. Multiple logistic regression was used to determine the independent predictors for each hepatitis and HIV co-infection. A p-value ≤ 0.05 was considered to be statistically significant.

### Ethical issues

Ethical clearance was obtained from the research and publications committee of the Muhimbili University of Health and Allied Sciences (MUHAS). Study participants provided informed written consent prior to enrolment. Patients found with hepatitis co-infection were started on triple ART regimen that included lamivudine, with efavirenz replacing nevirapine as a non-nucleoside anti-retroviral drug.

## Results

Two hundred and sixty HIV infected patients were recruited during the study period, of whom 70.0% were females. Table 1 summarises the characteristics of the study population. The overall mean age was 37.3 (± 9.5) years, ranging from 19 to 81 years. Males were significantly older, with a mean age of 41.4 years compared to females whose mean age was 35.6 years, p < 0.0001. The median CD4+ T-lymphocytes was 202 cells/μl.

**Table 1:** Socio-demographic characteristics of the study population (n = 260)

**Characteristic**	**Male (%)** **n = 78 **	**Female (%)** **N = 182**	**Total** **n = 260**	**P-value**
**Age group**				< 0.01
18 – 29	3 (3.8)	48 (26.3)	51 (19.6)	
30 – 39	38 (48.7)	87 (47.8)	125 (48.1)	
40 – 49	28 (35.9)	31 (17.0)	59 (22.7)	
50+	9 (11.5)	16 (8.8)	25 (9.6)	
				
**Marital Status**				0.04
Single	16 (20.5)	51 (28.0)	67 (25.8)	
Married	47 (60.3)	83 (45.6)	130 (50.0)	
Divorced	10 (12.8)	18 (9.9)	28 (10.8)	
Widowed	5 (6.4)	30 (16.5)	35 (13.5)	
				
**Education**				0.04
Primary & below	44 (56.4)	125 (68.7)	169 (65.0)	
Secondary	22 (28.2)	41 (22.5)	63 (24.2)	
Post Secondary	12 (15.4)	16 (8.8)	28 (10.8)	
				
**Baseline CD4+ count (cells/μl)**				
< 200	43 (56.6)	77 (46.1)	120 (49.4)	
200 – 350	19 (25.0)	27 (16.2)	46 (18.9)	
> 350	14 (18.4)	63 (37.7)	77 (31.7)	< 0.01
				
**WHO-HIV stage**				0.66
**I**	15 (19.2)	45 (24.7)	60 (23.1)	
**II**	20 (25.6)	47 (25.8)	67 (25.8)	
**III**	32 (41.0)	72 (39.6)	104 (40.0)	
**IV**	11 (14.1)	18 (9.9)	29 (11.2)	

The seroprevalence of HAV (anti HAV IgM), HBV(HBsAg), acute HBV(anti HBcAb) and HCV (anti HCV) were 3.1%, 17.3%, 2.3% and 18.1%, respectively (Table 2). Of the six patients with acute Hepatitis B infection (anti-HBc IgM antibody positive), two had occult HBV infection (anti HBc IgM positive but HBsAg negative), and the remaining four were positive for HBsAg. HBV carrier state, defined as being HBsAg positive and HBc IgM antibody negative, was present in 41 patients (15.8%). The prevalence of any of the four hepatitis markers was found to be 34.6% (Table 2).

**Table 2:** Sero-prevalence of viral hepatitis markers among the study population (n = 260)

**Marker**	**No (%) **	**95% CI**
**HAV-IgM antibodies**	8 (3.1)	1.3 – 6.0
**HBsAg**	45 (17.3)	12.9 – 22.5
**HBc IgM antibodies**	6 (2.3)	0.9 – 5.0
**HCV Ab**	47 (18.1)	13.6 – 23.3
**HBsAg & HBc IgM **	4 (1.6)	0.4 – 3.9
**HBsAg & HCV Ab**	10 (3.9)	1.9 – 7.0
		
**Any viral marker**	90(34.6)	28.9 – 40.7

Co-infection with both HBV and HCV (HBsAg and HCV Ab) occurred in 10 individuals (3.9%) while that of HAV and HBV (anti HAV IgM and HBsAg) occurred in two subjects (0.8%). None of the investigated patients was found to be co-infected with HAV and HCV. Similarly, none of the patients had all the three hepatitis viruses (HAV, HBV and HCV), (Table 2). There was neither an association between the occurrence of HBV and HCV nor between HAV and HBV.

The prevalence of HCV infection increased with age (χ_trend _= 4; p = 0.04), with the oldest age group (50+ years) having the highest proportion (Table 3). The prevalence of HBV infection among males (21.8%) was non-significantly higher than that among females (15.4%). Similarly, widowed and divorced subjects had the highest prevalence (39.7%) for HCV while currently married had the lowest prevalence of HCV (13.8%). Subjects who were never married had the highest prevalence (20.9%) of HBV infection. However, there was no statistically significant association between viral hepatitis infection and sex, marital status or education status (table 3). Furthermore, univariate analysis showed no association between the presence of any of the hepatitis viruses with blood transfusion, illicit drug use, uvulectomy and traditional or cosmetic scarification.

**Table 3:** Sero-prevalence of viral hepatitis markers according to demographic characteristics (n = 260)

**Characteristic**	**Number tested**	**Anti HAV-IgM +**	**HBsAg +**	**Anti HCV +**	**Any Hepatitis +**
	**N**	**n (%)**	**n (%)**	**n (%)**	**n (%)**
**Age**					
18 – 29	51	1 (2.0)	12 (23.5)	7 (13.7)	16 (31.4)
30 – 39	125	3 (2.4)	16 (12.8)	22 (17.6)	38 (30.4)
40 – 49	59	3 (5.1)	15 (25.4)	9 (15.3)	25 (42.4)
50+	25	1 (4.0)	2 (8.0)	9 (36.0)	11 (44.0)
					
P*-value		**	0.06	0.09	0.28
**Sex**					
Male	78	1 (1.3)	17 (21.8)	10 (12.8)	27 (34.6)
Female	182	7 (3.8)	28 (15.4)	37 (20.3)	63 (34.6)
					
P* value		0.442*	0.211	0.149	1
**Education**					
Primary & Below	169	4 (2.4)	29 (17.2)	35 (20.7)	62 (36.7)
Secondary & Above	91	4 (4.4)	16 (17.6)	12 (13.2)	28 (30.8)
					
P* value		0.456*	0.932	0.133	0.339
**Marital status**					
Never Married	67	2 (3.0)	14 (20.9)	12 (17.9)	24 (35.8)
Currently Married	130	5 (3.8)	23 (17.7)	18 (13.8)	41 (31.5)
Divorced/Widowed	63	1 (1.6)	8 (12.7)	17 (27.0)	25 (39.7)
					
P* value		**	0.460	0.084	0.522

**1. Key**: * = fishers' exact test ** = no test done due to small numbers. "**Any hepatitis**" = positivity of any of the viral markers tested antibodies

2. P* value compares the proportion on each marker by various sociodemographic categories

Table 4 shows that out of all patients with HAV infection 50% had nausea and 37.5% had vomiting. These proportions were significantly higher than corresponding proportions among patients without HAV infection. Moreover, patients with acute HBV (HBcAb IgM) were more likely to have jaundice (66.7%), hepatomegaly (66.7%), raised alanine aminotransferase {ALAT} (66.7%) or raised total bilirubin (66.7%). However, when clinical features and biochemical markers were considered together, only 17 out of 90 (18.9%) patients with any of the markers for hepatitis had jaundice (by history or examination), or hepatomegaly or elevated ALAT. Importantly, only 10.0% of the patients co-infected with hepatitis virus A, B or C co-infection had abnormal serum ALAT.

**Table 4:** Clinical characteristics of patients with viral hepatitis markers (n = 260)

	**HAV-IgM +ve**	**HbsAg +ve**	**HBcIgM +ve**	**HCV-ab +ve**
**Clinical feature, n (%)**				
Fever	3 (37.5)	17 (37.8)	2 (33.3)	18 (38.3)
Nausea	4 (50.0)*	5 (11.1)	1 (16.7)	7 (14.9)
Vomiting	3 (37.5)*	6 (13.3)	1 (16.7)	4 (8.5)
Jaundice	0	5 (11.1)	4 (66.7)*	4 (8.5)
Hepatomegaly	0	5 (11.1)	4 (66.7)*	3 (6.4)
				
**Biochemical characteristics, n (%)**				
Elevated ALAT	1 (12.5)	4 (8.9)	2 (33.3)*	2 (4.3)
Elevated Total bilirubin	0	5 (11.1)	4 (66.7)*	5 (10.6)
				
**Immunological characteristic**				
Mean CD4+ T-cells (cells/μl)	113	198	134	222

* = P < 0.05, when compared with those having no corresponding marker

Table 4 also shows that the mean CD4+ T-lymphocyte counts among patients co-infected with either HAV, or HBV, or HCV were not significantly different from the mean values among patients without the corresponding hepatitis co-infection.

A set of variables was selected to search for predictors of the viral hepatitis infections including, age, sex, blood transfusion, scarification, nausea and vomiting, jaundice, palpable hepatomegaly and the presence of other viral hepatitis infection. Significant predictors of co-infection with the respective viral hepatitis are summarised in table 5. Presence of nausea, history of prior blood transfusion, and presence of jaundice were the independent predictors of HAV, HBV and acute HBV co-infections respectively.

**Table 5:** Likelihood of viral hepatitis co-infection using clinical features presented as odds ratios (OR) with 95% confidence intervals (CI) (n = 243)*

**Outcome**	**Predictor**	**OR (95% CI)**	**p-value**
HAV	Nausea	4.18 (1.46 – 11.96)	**< 0.01**
HBsAg	Blood transfusion	2.59 (1.09 – 6.15)	**0.03**
HBcAb	Jaundice	41.07 (6.78 – 248.89)	**< 0.01**

*CD 4+ T lymphocyte counts for 17 patients were lost and could not be traced.

## Discussion

This study reveals a high burden of hepatitis viral co-infection among HIV infected, ART naïve patients who sought care at a tertiary public health facility in Tanzania, with more than a third of the study participants being co-infected.

HCV was the most prevalent infection (18.1%) which is in keeping with the findings of Telatela et al [6] who documented HCV sero-prevalence of 13.8% among HIV infected children in the same institution [6]. This prevalence is higher than that reported among among blood donors at the same hospital [7-9] probably due to the fact that all our subjects were HIV-infected and that HCV has similar transmission modes as HIV. The lower prevalence of HCV reported by Wadell et al. [8] could also be due to the fact that they used an HCV RNA assay, which is a more stringent assay [10]. The increase in the prevalence of HCV infection with age that was observed is a reflection of a cumulative risk with time [11].

We found a higher prevalence of HBsAg (17.3%) compared to a study conducted among blood donors (8.8%–11.0%) at the same setting [7,9]. Three main reasons are postulated; firstly, the shared modes of transmission between HIV and HBV [12]. Secondly, is the known phenomenon of reactivation of HBV in the setting of HIV immunodeficiency [13] and lastly, the higher rates of childhood horizontal HBV transmission in sub-Saharan Africa [14]. It is important to note two subjects with anti HBc IgM antibodies but negative HBsAg who may have had occult HBV viremia as demonstrated previously in HIV subjects [15]. Notably occult HBV infection is likely to be missed diagnosis when HBsAg is employed as a routine screening test, which may consequently lead to silent liver damage.

The prevalence of HAV-IgM (3.1%) reported in this study is much lower than that of 41.0% found in a Kenyan study [16]. The difference is most probably due to the fact that the latter study investigated patients who already had clinical hepatitis as evidenced by jaundice.

About 4% of our study population had dual HBV/HCV infection evidenced by co-existence of HBsAg and anti-HCV total antibodies. However, the occurrence of the two viruses (HBV and HCV) was not significantly associated, corroborating the findings of previous studies done in the country [6,7].

In this study, the association of hepatitis viral co-infection with serum alanine aminotransferase (ALAT) as well as clinical features was very limited. Only 10% of HIV/hepatitis co-infected patients had elevated ALAT and clinical features suggestive of acute liver disease were insignificantly associated with hepatitis co-infection. The asymptomatic pattern of co-infection in our study population would probably be due to the fact that we detected less subjects with acute hepatitis (HAV-IgM 3.1%, HBsAg 2.3%) compared to those with chronic HBV and HCV co-infection. The paucity of signs and symptoms of liver damage has been documented in HIV patients with chronic viral hepatitis co-infection [17,18] and has been attributed to decreased hepatocellular injury associated with the HIV-induced immunodeficiency [19].

In this study, except for the association between age and HCV infection, demographic characteristics were not associated with the occurrence of any of the investigated hepatitis infections. Our results show that HCV co-infection was associated with increasing age, which may reflect a cumulative risk over time as previously reported by Kondili [11]. Notably, we observed no statistically significant association between the occurrence of the either HBsAg or HCV and sex and marital status, which is in keeping with other studies conducted in the country [20].

In this study there was no association between absolute CD4+ T-lymphocyte count and the presence of hepatitis co-infection. Indeed the association between hepatitis viral co-infection and CD4+ T lymphocytes count is currently a subject of debate, with some studies showing no existence of a relationship [21] and others showing the association [22]. The lack of association is probably due to the impaired qualitative response to hepatitis virus in HIV infection rather than impairment in the absolute numbers of CD4+ T-lymphocytes [23]. However, the lack of association in our study could also be due to that fact that most of our study patients had low CD4 counts, (median < 202 cells/mm^3^) at baseline.

Evidently from this study is the fact that most (> 80%) of the patients with hepatitis viral co-infection, presented with no specific liver damage-related signs and biochemical changes, emphasizing the need for routine screening of viral markers in HIV patients. This is currently not part of the Tanzanian HIV care and treatment guidelines [24]. Secondly, there are implications for the choice of an appropriate regimen for the hepatitis co-infected patients that should include two active anti HBV agents to avoid viral mutations and thus enhance outcome [25,26]. The, current first line ART regimen in Tanzania (stavudine, lamivudine and nevirapine or efavirenz) [24] does not sufficiently cover for HBV [25]. Moreover, a prolonged use lamivudine against HIV infection in undiagnosed viral hepatitis infection may result in resistant HBV mutants [27]. Flexibility in the regimen under such circumstances to include appropriate drugs to take care of HBV or HCV co-infections should be considered. In addition, it is likely that the undiagnosed cases of hepatitis co-infected patients may be kept on nevirapine based ART combinations leading to nevirapine hepatotoxicity [28], and thus defeating the national objective of improving the quality of life of these patients [24]. Finally, patients with HIV/Hepatitis co-infection may complicate ART with an immune reconstitution syndrome (IRIS), which may be confused with drug toxicity or acute hepatitis infection. It is therefore important for health care workers to be cognisant of these facts to improve the care of patients infected with HIV

## Conclusion

HBV and HCV infections are relatively common among Tanzanian HIV patients and should be considered during baseline work-up of HIV patients in order to provide appropriate ART. The limited predictive value of clinical features and biochemical markers indicate that care and treatment centers should be equipped with laboratory facilities to diagnose hepatitis viral co-infection. Scaling up of ART services in the country should go hand in hand with reviewing HIV treatment guidelines to cater for HBV/HCV co-infection.

## Competing interests

The authors declare that they have no competing interests.

## Authors' contributions

TJN designed the study, conducted the interviews and clinical examinations. MB assisted study design and supervised interviews and clinical examination. MIM assisted study design and supervised laboratory work. Finally, all authors participated in the preparation of the manuscript, read and approved the final manuscript.

## Pre-publication history

The pre-publication history for this paper can be accessed here:


